# Supplementary material for: Compression-sensitive smart windows: inclined pores for dynamic transparency changes
Source: Nat Commun. 2024 Sep 14;15:8074. doi: 10.1038/s41467-024-52305-6 (PMC11401924; doi:10.1038/s41467-024-52305-6)
Supplement: Supplementary file 1 — Supplementary Information [file 41467_2024_52305_MOESM1_ESM.pdf]

Supplementary Materials for

Compression-Sensitive Smart Windows: Inclined Pores for Dynamic Transparency Changes

Haomin Chen, Gunho Chang, Tae Hee Lee, Seokhwan Min, Sanghyeon Nam, Donghwi Cho, Kwonhwan Ko, Gwangmin Bae, Yoonseong Lee, Jirou Feng, Heng Zhang, Jang-Kyo Kim, Jonghwa Shin, Jung-Wuk Hong* & Seokwoo Jeon*

Corresponding author: E-mail: j.hong@kaist.ac.kr (J.W. Hong); jeon39@korea.ac.kr (S. Jeon)

The PDF file includes:

Supplementary Text
Supplementary Figure 1 to 17
Supplementary Table 1
Supplementary References (1-3)

Other Supplementary Materials for this manuscript include the following:

Supplementary Movies 1 to 3
Source Data

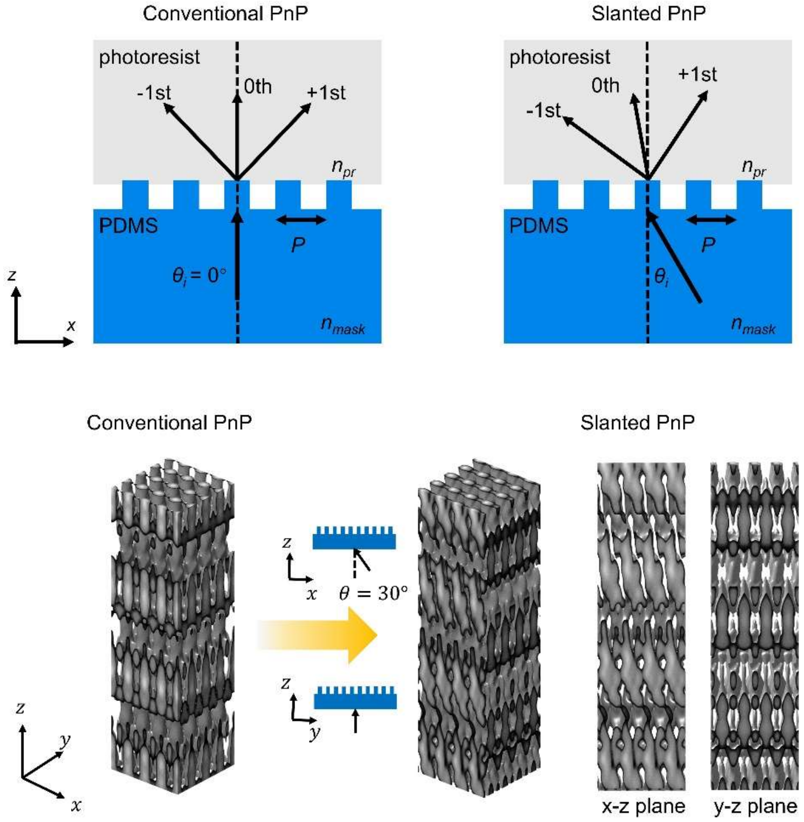

Supplementary Figure 1

Schematics illustrating (a) the symmetric diffracted beams in the conventional PnP and (b) the asymmetric beams in the slanted PnP. (c) Simulated structures fabricated *via* conventional PnP using normal incident light and slanted PnP using slanted incident light.

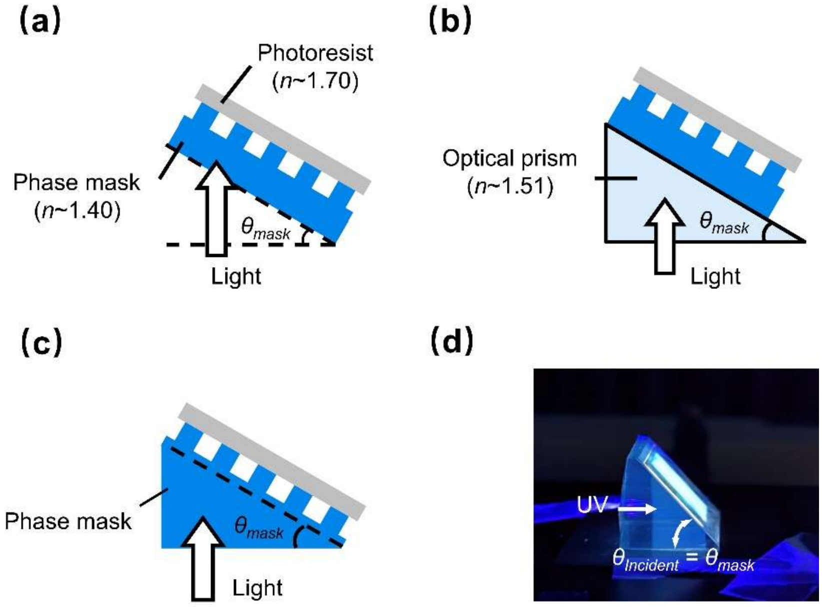

Supplementary Figure 2.
Schematics illustrating different designs of phase masks for slanted exposure: (a) normal phase mask tilted in the air, (b) normal phase mask placed atop an optical prism, and (c) slanted phase mask. (d) Digital photograph of the prism-shaped phase mask with an incident angle of 45°.

Because of the refraction at the air/mask interface (Supplementary Figure 2a), the incident angle was much reduced from the initial θ_{mask}, making the first design ineffective. In the second design (Supplementary Figure 2b), the optical prism made of BK7 glass had a refractive index of 1.51, which mismatched with that of a PDMS-made mask. As a result, reflection and scattering occurred at the prism/mask interface, leading to hazing of the light and, thus, a compromised optical contrast. On the contrary, the monolithic phase mask with a prism shape avoids additional interfaces, enabling better incident angle control (Supplementary Figure 2c and 2d).

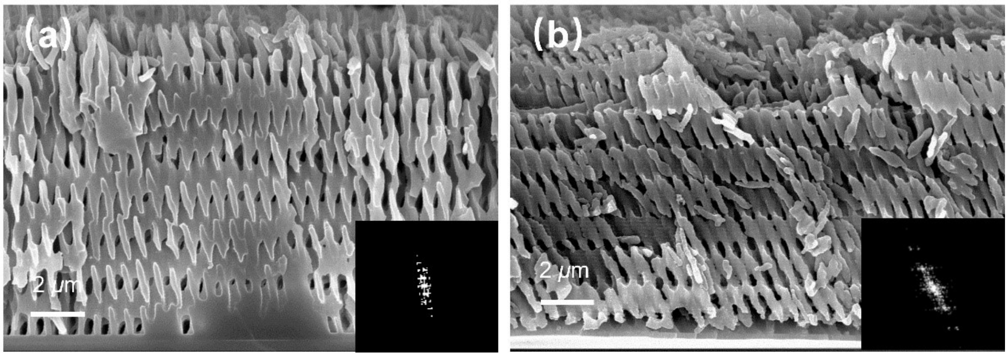

Supplementary Figure 3.

SEM images of the inclined 3D structure were prepared at incident angles of (a) 15° and (b) 45°. Insets show the Fast-Fourier Transform frequency domain patterns of the corresponding SEM images for measurement of the structural inclination.

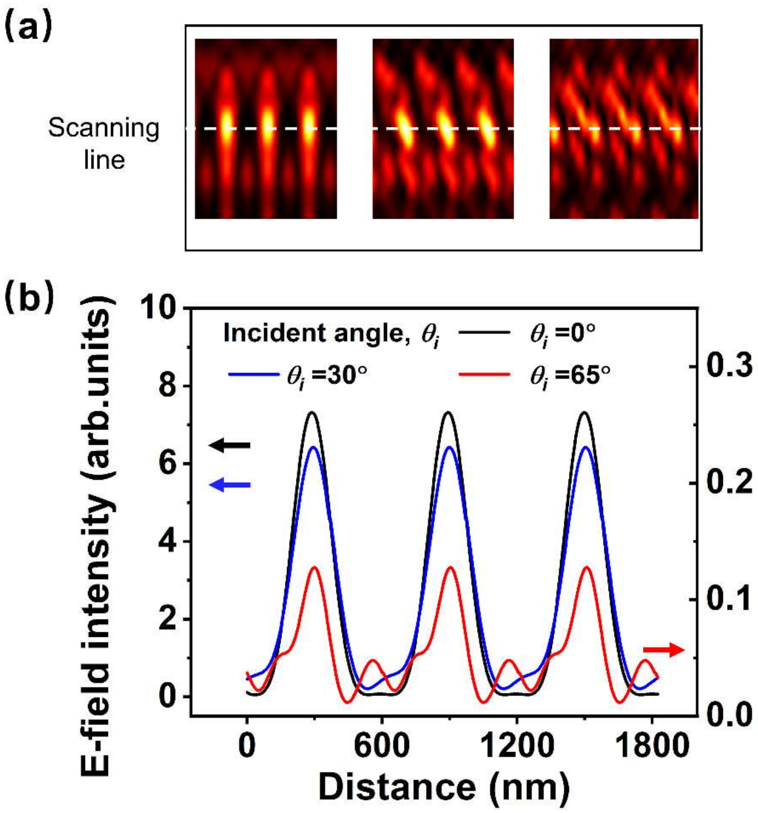

Supplementary Figure 4.

(a) Simulated intensity distributions of the diffraction patterns and (b) E-field intensity along the scanning line for incident angles of 0°, 30°, and 65°.

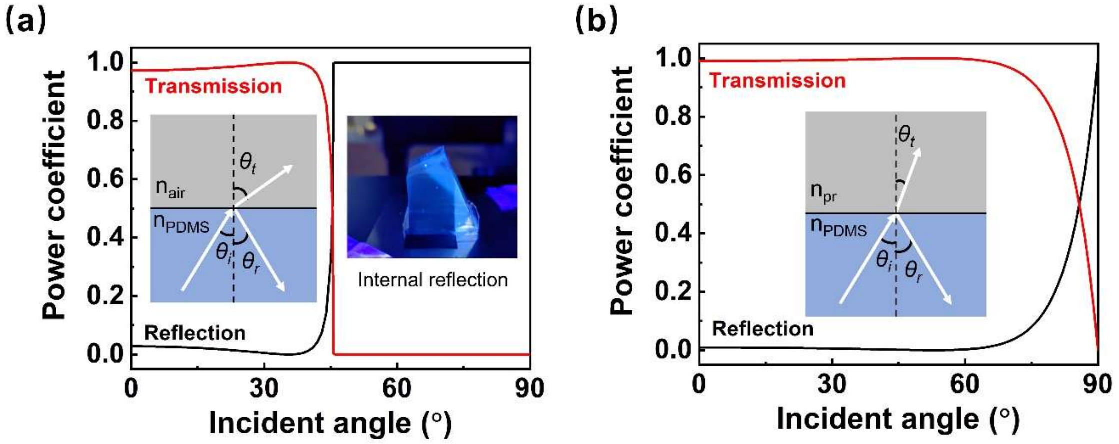

Supplementary Figure 5.

Calculated power coefficients of transmission and reflection as a function of incident angle at the simplified (a) mask/air interface and (b) mask/photoresist interface without considering the gratings. Insets: schematics showing the light paths and digital photograph of a 65° mask with strong internal reflection.

According to the Fresnel equations,

$$R = \left|\frac{n_2 \cos \quad _i - n_1 \cos\theta_t}{n_2 \cos \quad _i + n_1 \cos\theta_t}\right|^2 \tag{S1}$$

$$T = 1 - R \tag{S2}$$

where R and T are the power coefficients of reflection and transmission, n_1 and n_2 are the refractive indices of the materials where light is incident and transmitted, and θ_i and θ_t are the incident angle and refracted angle, respectively. The critical angle required for most of the incident light to be reflected at the simple mask/air interface was found to be 45.6°. In contrast, the power coefficient of the transmitted light remained over 98% until θ_i became larger than 68° at the mask/photoresist interface. This means the conformal contact between the photoresist and mask is critical to guarantee the patterning quality.

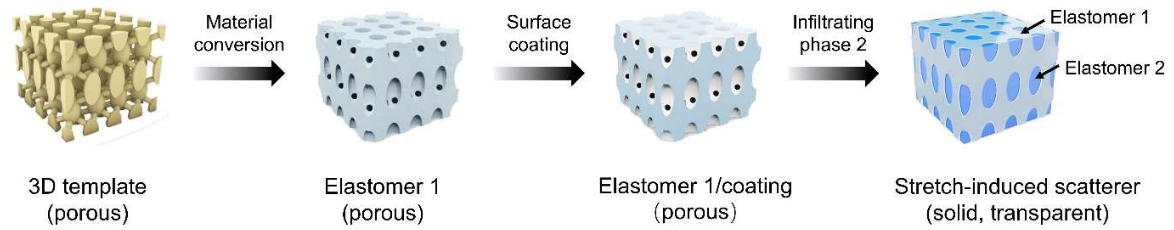

Supplementary Figure 6.
Schematics illustrating the fabrication of in-plane stretch-mode scatterers.

The device size can be strongly restricted by the process required to produce the intermediate layer, which was necessary in the previous studies, as shown in Supplementary Figure 6 (20, 27). Therefore, the fabrication process in this work, which eliminates the need to generate the intermediate layer, shows important progress in developing advanced scatterers that are easy to scale up.

To further scale up the fabrication, integrating the PnP technique with a continuous exposure method may realize 3D patterning in a roll-to-roll form and break the current limitation of production dimension and time. By mass production, the 3D patterning would become cost-effective and can be widely exploited in various applications. This may involve systematic process optimization and re-design of the phase mask geometry, where issues include enlarging the phase mask and light beam, guaranteeing the consistent alignment between the mask and photoresist, and ensuring the exposure dose across the large-area sample.

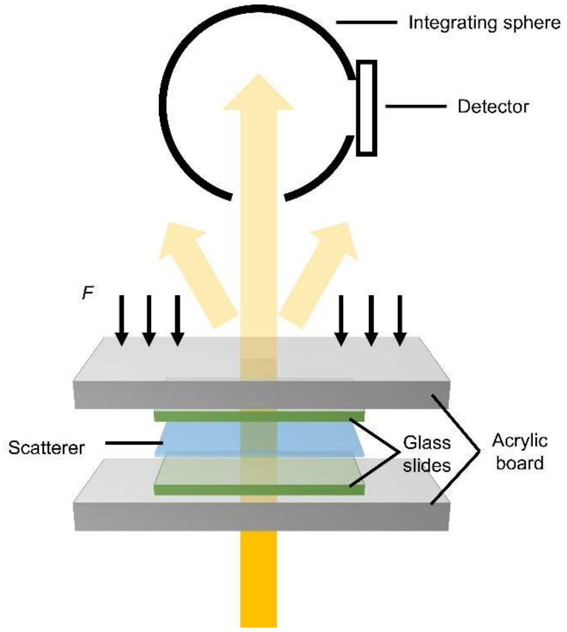

Supplementary Figure 7.
Schematic illustrating the setup used to characterize the transmittance of the scatterer under compression.

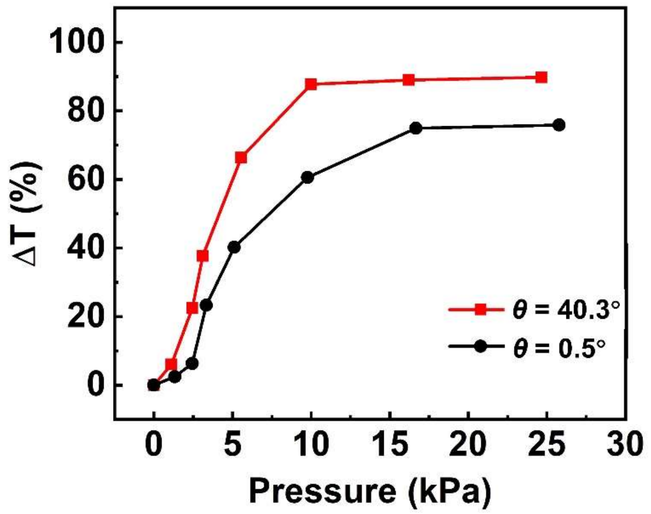

Supplementary Figure 8.
Normal transmittance changes of the 3D scatterers with different inclined angles as a function of applied normal pressure.

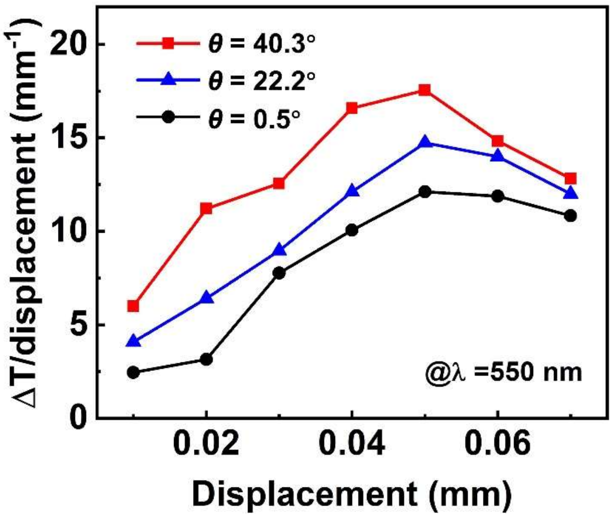

Supplementary Figure 9.

Optical modulating sensitivity of the 3D scatterers as a function of through-thickness compressive displacement.

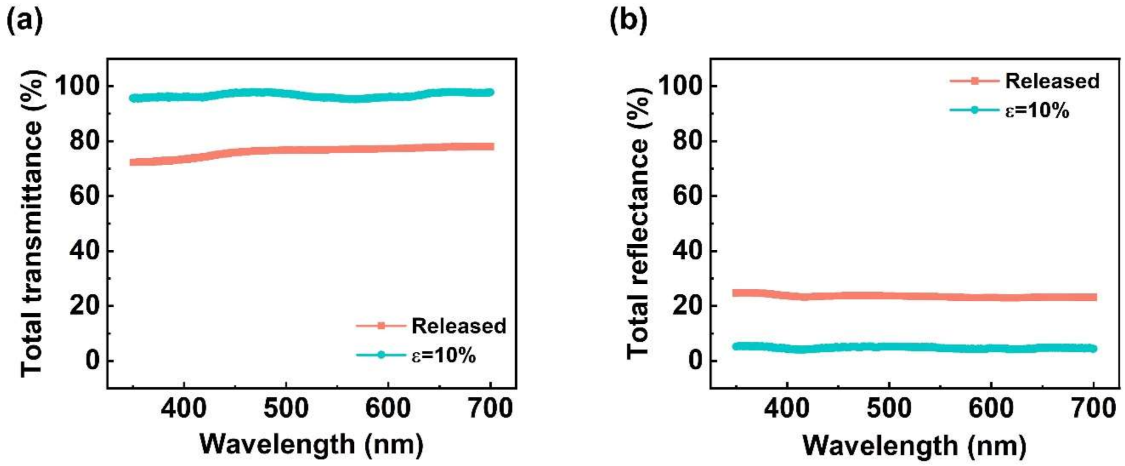

Supplementary Figure 10.

(a) Total transmittance and (b) total reflectance of the optimized 3D scatterer before and after the application of 10% total strain.

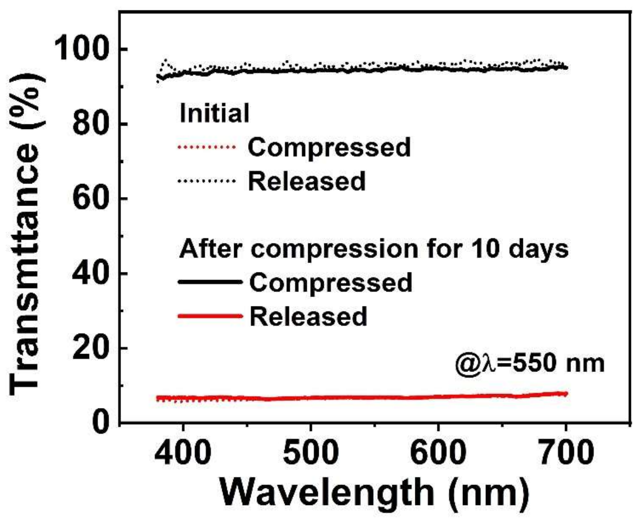

Supplementary Figure 11.

Durability tests of the 3D scatterer: transmittance of the optimized 3D scatterer before and after continuous compression for ten days.

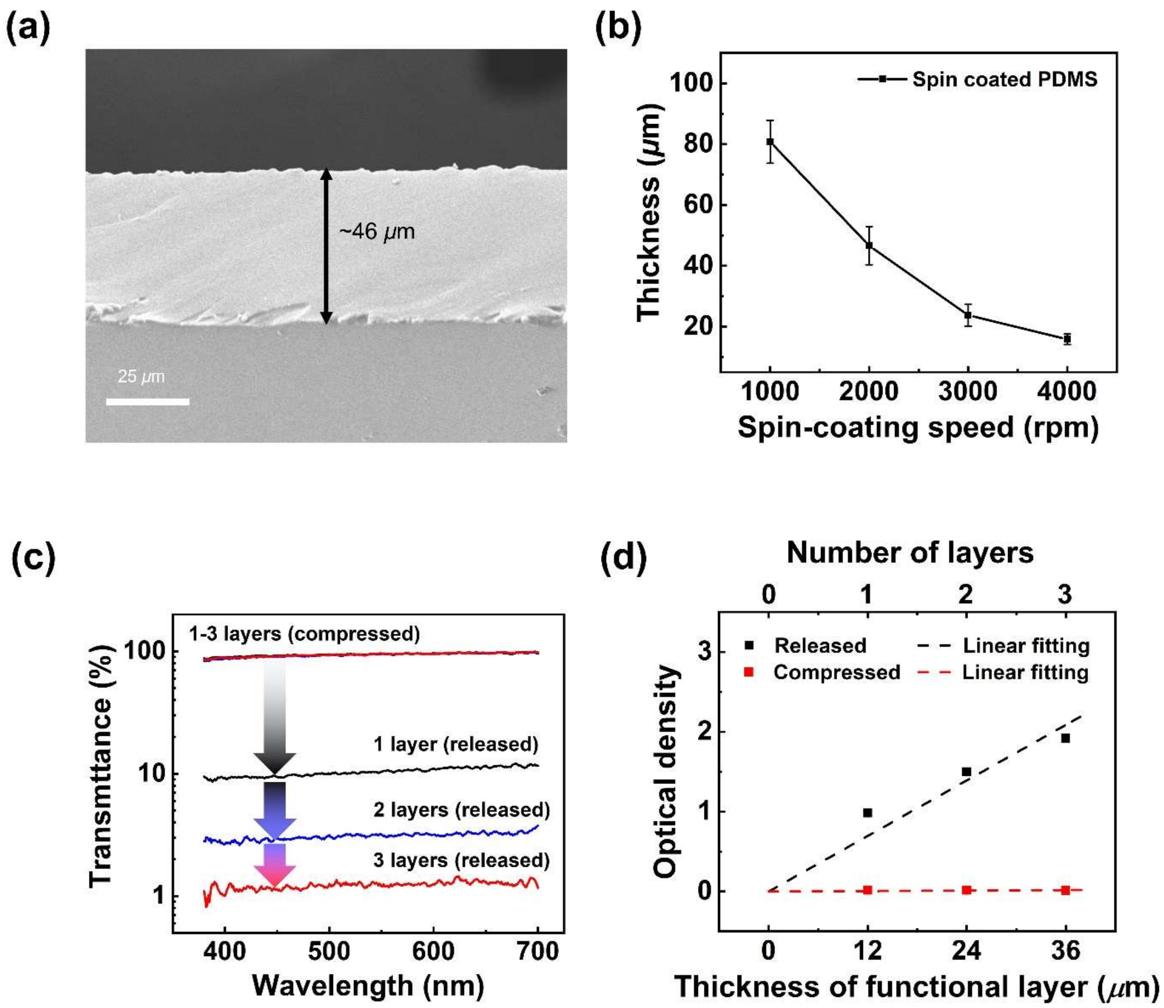

Supplementary Figure 12.

(a) SEM image showing the thickness of the spin-coated PDMS. (b) The thickness of the spin-coated PDMS as a function of spin-coating speed. Error bars indicate standard deviations. (c) Normal transmittance of the stacked ultrathin 3D scatterers in the released and compressed states. (d) Optical density of the stacked ultrathin 3D scatterers as a function of functional layer thickness and the number of stacked layers.

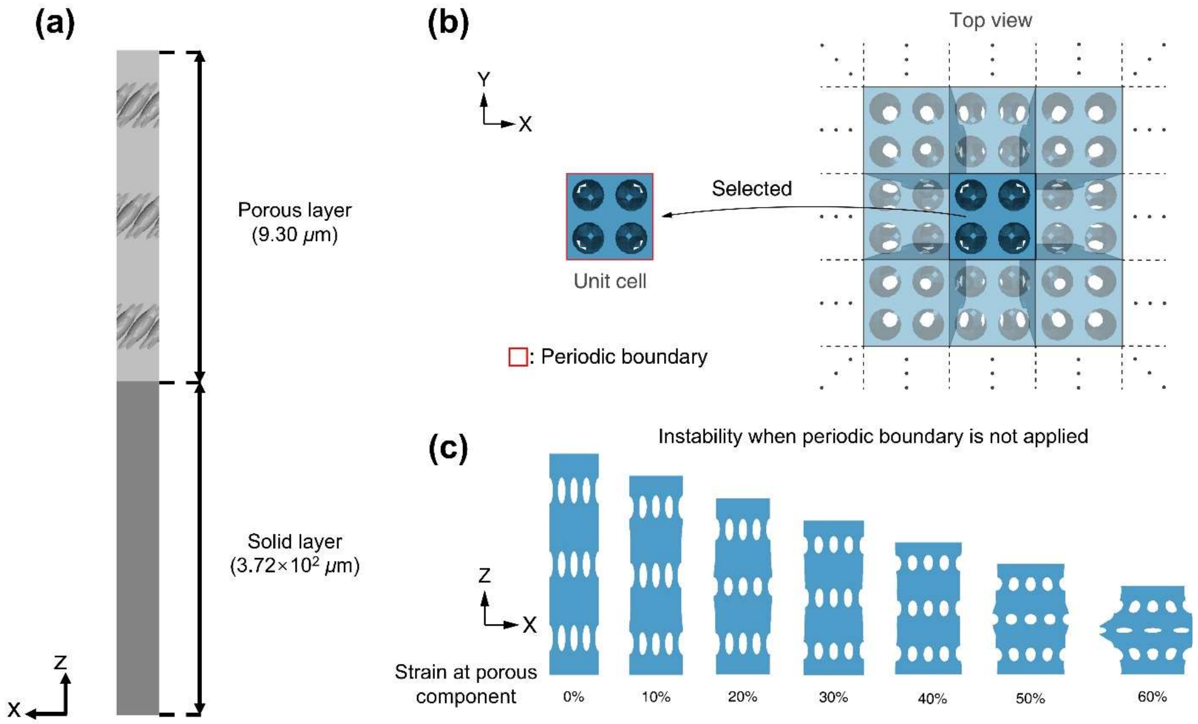

Supplementary Figure 13.
(a) Description of the FEA model. (b) Periodic boundary conditions applied to a unit cell selected from the interior of the material. (c) Buckling behavior of the porous component when no periodic boundary is applied.

In the finite element numerical simulation, a periodic boundary condition was applied to the unit cell, as illustrated in Supplementary Figure 13b, to eliminate edge effects such as buckling (Supplementary Figure 13c). This condition constrains the boundary nodes on each face to exhibit the same displacements, effectively simulating the behavior of the developed material with a periodic porous structure under a compression load applied in the -z direction, ensuring stable responses of the simulation.

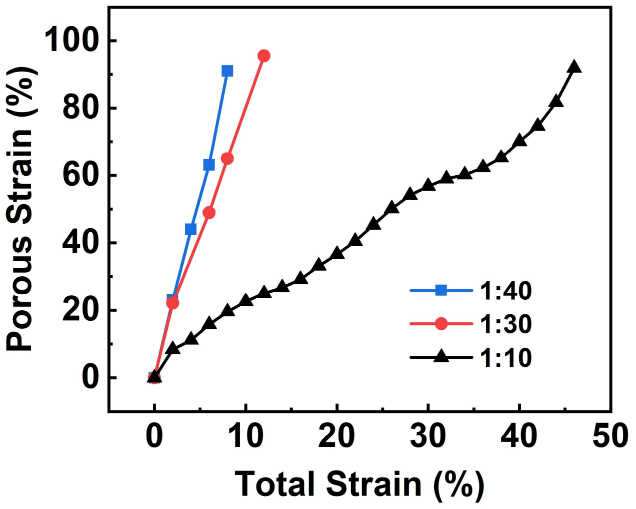

Supplementary Figure 14.

Simulated porous strains of the models with different thickness ratios of porous to solid components.

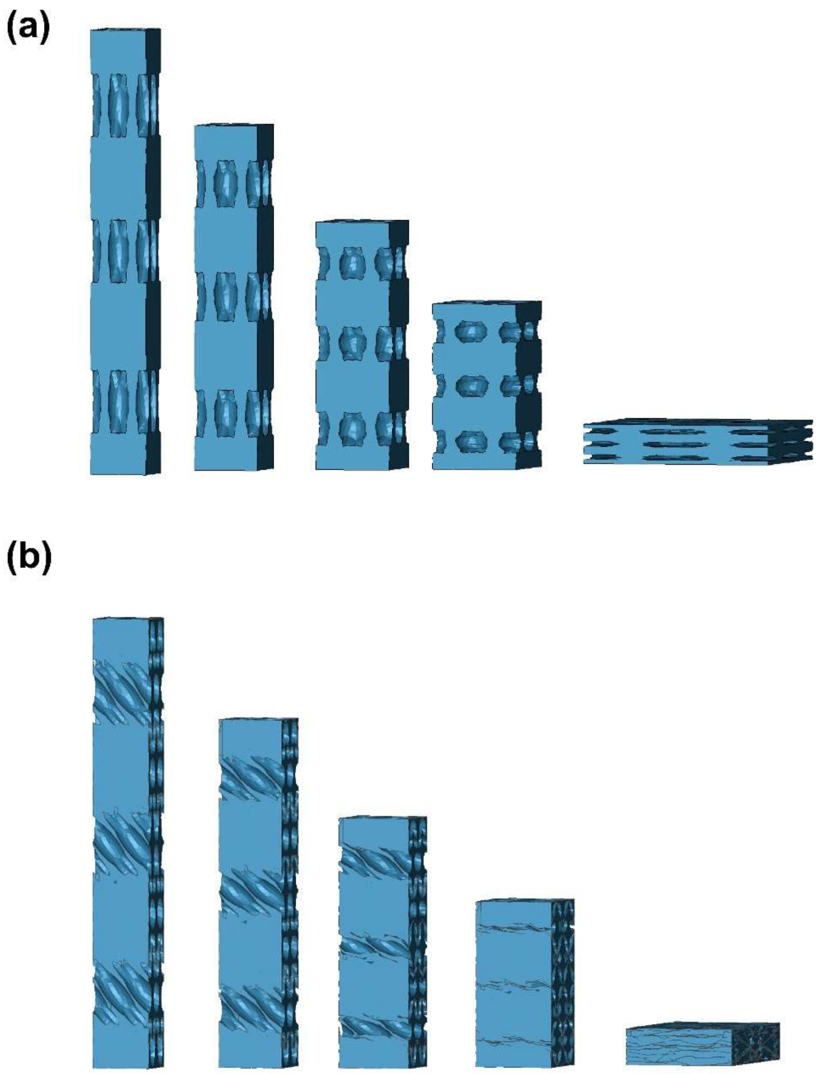

Supplementary Figure 15.

Deformations of the porous functional layer of the model with (a) vertically aligned pores and (b) inclined pores at 40 degrees when total compressive strains of 0%, 2%, 4%, 6%, and 8% are applied.

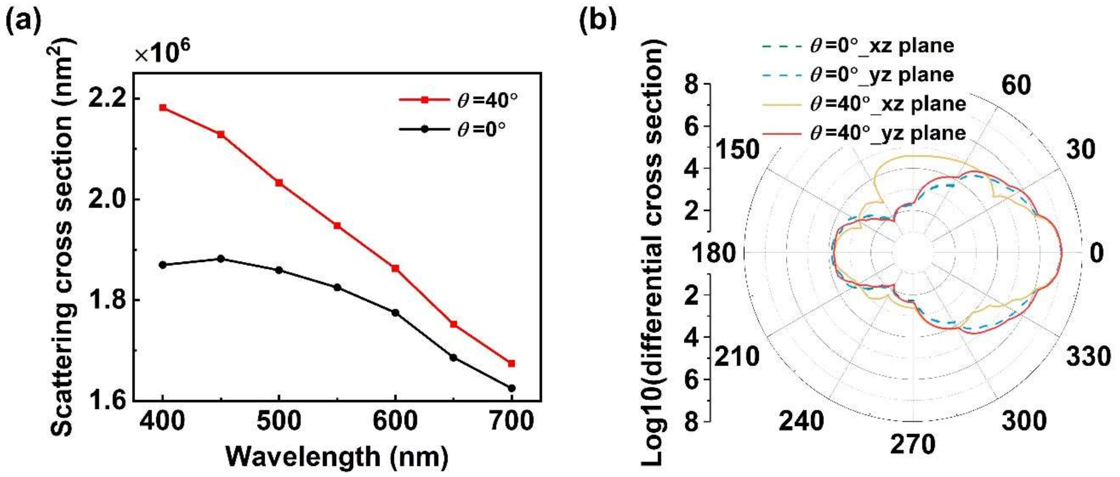

Supplementary Figure 16.

(a) Scattering cross-section spectra of the models with different pore angles of 0° and 40°. (b) Differential scattering cross sections of the models with vertically aligned and inclined pores.

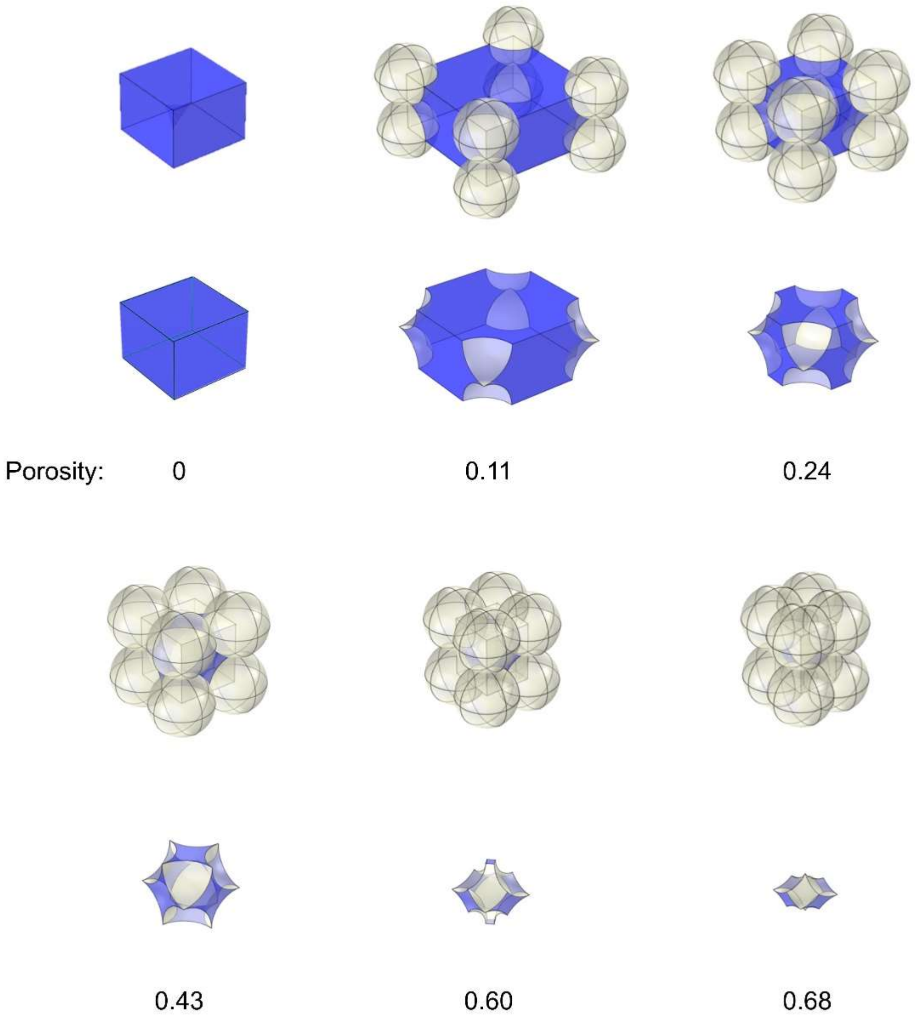

Supplementary Figure 17.
Models used in the FEA simulations to investigate the influence of porosity and pore configurations on the additional space requirement under through-thickness compression.

Supplementary Table 1.
Constituent material properties.

PDMS (Nanostructure)	
Density (kg/$\mathbf{m^3}$)	965
Young's modulus (MPa)	1.1
Poisson's ratio	0.499
Yield strength (MPa)	0.7
**Steel (Plate)**	
Density (kg/$\mathbf{m^3}$)	7,850
Young's modulus (MPa)	200,000
Poisson's ratio	0.3

Supplementary Text

FEA simulations

LS-DYNA, a nonlinear finite-element analysis (FEA) platform with a large library of material models and element formulations, was employed to analyze effective strains under compression. FEA is considered an appropriate method for predicting physical phenomena in the field of engineering (1). Three porous nanostructures were modeled, containing pores with different inclined angles of 0, 20, and 40 degrees against the vertical direction of the functional layer. The 3D nanostructure consisted of a porous functional layer with 2 × 2 × 3 unit cells placed on top of a solid layer, and a rigid plate was positioned on the functional layer for loading.

Although a thickness ratio of 1:50 was used in experiments, a slightly larger ratio of 1:40 was employed in the simulation to reduce the computational cost. It was found that there was a negligible increase in net strains in the porous layer under compression when the ratio was changed from 1:30 to 1:40 (Supplementary Figure 14). Thus, 1:40 was considered adequate to represent the experimental condition of 1:50 without compromising the accuracy of the results.

We carefully chose material models for the simulations to enhance calculation accuracy (Supplementary Table 1). A bilinear elastic-plastic model which is widely used for simulating isotropic and kinematic hardening plasticity was used for PDMS, and an elastic model was employed for the plate due to the significantly larger elastic modulus of the plate and negligible deformation compared to those of the nanostructure.

We discretized the functional layer using tetrahedral solid elements to accurately represent its complex geometry and allow large deformations (2), while hexahedral solid elements were used for the plate and solid layer to reduce computational time. To prevent penetration between the plate and the nanostructure under compressive loads, we applied a contact condition to their surfaces. The central axis of the nanostructure, the bottom surface of the solid layer, and the plate were fully constrained under compression, except in the longitudinal direction. To enable uniform deformations along the sides of the nanostructure, we applied a periodic boundary condition along the whole in-plane direction. Compressive loads were applied both to the plate and bottom surface of the nanostructure at a strain rate of 3.3×10^{-4} s^{-1}. All simulations were performed on a multi-core system equipped with a Dual Intel ® Xeon® CPU (E5-2687W v4) running at 3.00 GHz of 48 threads with 128 GB RAM.

Simulation of transmittance

The normal transmittance of the scatterer was modeled using a bi-level simulation framework. First, the scattering cross section and the scattering phase function of an individual unit cell were computed using the full-wave FDTD simulation with non-periodic boundary conditions. These quantities were then inputted into an in-house Monte Carlo ray-tracing simulation (3). A total of 1,000,000 photon trajectories were used per wavelength. Once a photon entered the film, its path length was sampled from an exponential distribution with ℓ as the mean free path, which is given by:

$$\ell = 1/(\rho C_{sca}) \tag{S3}$$

Here, ρ is the unit cell number density and C_{sca} is the scattering cross section. After propagation along the randomly sampled path length, the photon was scattered in a direction determined probabilistically by the unit cell scattering phase function. The propagation and scattering steps were repeated until the photon exited the film on either side. The polar and azimuthal angles for the exit direction were subdivided into 2,178 regions with equal solid angles and the number of photons exited through each region was collected to determine the angular reflectance and transmittance spectra.

The differential scattering cross section was computed through full-wave FDTD simulations by isolating the scattered field using the total-field scattered-field (TFSF) source, projecting it to the far field, and resolving its angular distribution. The total scattering cross section was then obtained by integrating the differential scattering cross section over a spherical surface in the far field.

Supplementary References

1. Bathe, Finite Element Procedures (2nd ed.), Prentice Hall (MIT Center for Advanced Engineering study, 2014).
2. Wu, R. B. & Itoh, T. Hybrid finite-difference time-domain modeling of curved surfaces using tetrahedral edge elements. *IEEE Transactions on Antennas and Propagation* **45**, 1302–1309 (1997).
3. Hwang, V. Designing angle-independent structural colors using Monte Carlo simulations of multiple scattering. *Proceedings of the National Academy of Sciences* **118**, 2015551118–

2015551118 (2021).
