# Supplementary material for: Compression-sensitive smart windows: inclined pores for dynamic transparency changes
Source: Nat Commun. 2024 Sep 14;15:8074. doi: 10.1038/s41467-024-52305-6 (PMC11401924; doi:10.1038/s41467-024-52305-6)
Supplement: Supplementary file 3 — Description of Additional Supplementary Files [file 41467_2024_52305_MOESM3_ESM.pdf]

Description of Additional Supplementary Files

Supplementary Movie 1. Response time and area expansion.

Supplementary Movie 2. Inclined pores vs normal pores.

Supplementary Movie 3. Compress vs stretch.
